# SeqWiz: a modularized toolkit for next-generation protein sequence database management and analysis

**DOI:** 10.1186/s12859-023-05334-9

**Published:** 2023-05-17

**Authors:** Ping Zhang, Min Wang, Tao Zhou, Daozhen Chen

**Affiliations:** 1 Research Institute for Reproductive Medicine and Genetic Diseases, The Affiliated Wuxi Maternity and Child Health Care Hospital of Nanjing Medical University, Wuxi, 214002 China; 2 Wuxi Maternity and Child Health Care Hospital, Wuxi School of Medicine, Jiangnan University, Wuxi, China

**Keywords:** Protein sequence database, Sequence format, Sequence processing, Sequence variation, Small open reading frame

## Abstract

**Background:**

Current proteomic technologies are fast-evolving to uncover the complex features of sequence processes, variations and modifications. Thus, protein sequence database and the corresponding softwares should also be improved to solve this issue.

**Results:**

We developed a state-of-the-art toolkit (SeqWiz) for constructing next-generation sequence databases and performing proteomic-centric sequence analyses. First, we proposed two derived data formats: SQPD (a well-structured and high-performance local sequence database based on SQLite), and SET (an associated list of selected entries based on JSON). The SQPD format follows the basic standards of the emerging PEFF format, which also aims to facilitate the search of complex proteoform. The SET format is designed for generating subsets with with high-efficiency. These formats are shown to greatly outperform the conventional FASTA or PEFF formats in time and resource consumption. Then, we mainly focused on the UniProt knowledgebase and developed a collection of open-source tools and basic modules for retrieving species-specific databases, formats conversion, sequence generation, sequence filter, and sequence analysis. These tools are implemented by using the Python language and licensed under the GNU General Public Licence V3. The source codes and distributions are freely available at GitHub (https://github.com/fountao/protwiz/tree/main/seqwiz).

**Conclusions:**

SeqWiz is designed to be a collection of modularized tools, which is friendly to both end-users for preparing easy-to-use sequence databases as well as bioinformaticians for performing downstream sequence analysis. Besides the novel formats, it also provides compatible functions for handling the traditional text based FASTA or PEFF formats. We believe that SeqWiz will promote the implementing of complementary proteomics for data renewal and proteoform analysis to achieve precision proteomics. Additionally, it can also drive the improvement of proteomic standardization and the development of next-generation proteomic softwares.

**Supplementary Information:**

The online version contains supplementary material available at 10.1186/s12859-023-05334-9.

## Background

Protein sequence database is the basis of shotgun proteomic analysis. It provides a collection of reference protein sequences to interpret the raw spectra. Thus, the completeness and accuracy of a database directly determine the quality of proteomic results. However, it should be noted that the current designs of sequence database and search engine are intrinsically imperfect to uncover the complex features of protein sequences. It is well known that the translated protein precursors usually undergo various processes (such as the removal of initiator methionine or the cleavage of peptide chains) before becoming fully matured in vivo. Additionally, due to the occurrence of sequence variations, mutations and post-translational modifications (such as phosphorylation and acetylation), there may be many concurrent forms for a reference protein sequence in the real-world samples. Here, we quotes and extends the terminology of “proteoforms” [1] to represent the complex forms of protein sequences. Most of the current sequence databases only adopt the full-length reference sequences for database search, which will definitely decrease the interpreting ratio of raw spectra and protein coverage. This issue may be partially due to the persistence of the text-based format of sequence: FASTA. Recently, the Proteomics Standards Initiative (PSI) has proposed the PSI extended FASTA format (PEFF) to facilitate the search of known sequence variants and modifications [2]. The PEFF format defines and annotates sequence processing, modifications, variants and proteoforms as key-value pairs in the header line for each sequence record. It also provides a reference standard (including controlled vocabularies) for the development of precision proteomic sequence databases and softwares. However, from a programmer’s point of view, the creation and parsing of PEFF is still complex and low-efficient due to pure text based recording of a large number of dictionary objectives.

Many integrated proteomic softwares such as MaxQuant [3] and mascot [4] have already supported a few features of sequence processes (such as the effects of initiator methionine and protein N-terminal acetylation), which are easily to be prepared for database search. The up-to-date versions of several modernized search engines, such as SPIDER [5] and Comet [6], are also capable to detect possible modification or mutation sites. However, the direct search of truncated peptides or varied amino acids will greatly increase the computational cost and false discovery rates. Thus, both of the architectures of sequence database and search engine are in urgent need to be updated to meet the complexity of proteoform analysis. Moreover, the general workflow of proteomic data analysis consist of multiple steps including database preparation, raw data processing, database search, protein grouping and quantification. And the variety of experimental methods and research objectives also make it hard to develop a one-stop software for solving all problems. The development of modularized and standalone softwares will greatly improve the flexibility of their applications. For example, the Andromeda was separated from the MaxQuant platform and can be used as a standalone search engine [7]. There are also many specialized packages for discovering complex modifications (such as pLink for cross-linked peptides [8]). However, there still lacks of specialized tools for complex sequence database preparation and analysis.

## Implementation

**Fig. 1:**
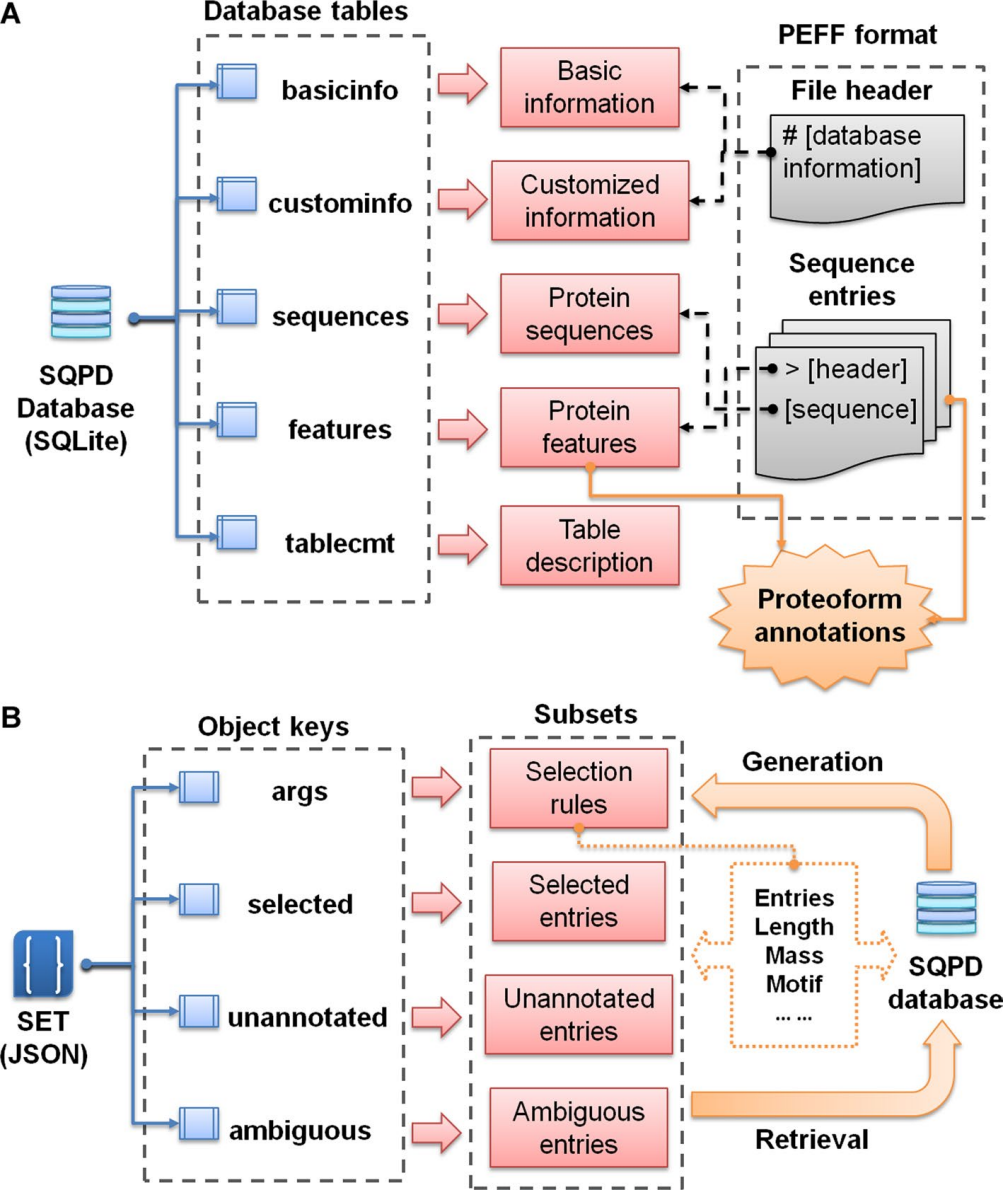
The design and structure of SQPD and SET format. **A**. The table structures and the corresponding contents of SQPD format. The right part of the diagram also indicates the relations between SQPD and PEFF. **B**. The object keys and the corresponding contents of SET format. The right part of the diagram also shows the paths for generation or retrieval of a SET file based on the SQPD

Considering the above issues, we aim to develop a novel toolkit, named as SeqWiz, for constructing next-generation sequence databases and performing proteomic-centric sequence analyses. To support the complex requirements of next-generation sequence databases, we first proposed two new derived formats: SQPD (a protein sequence database based on SQLite), and SET (an associated list of selected entries based on JSON). The SQPD format follows the basic structures and standards of the emerging PEFF format, which also aims to facilitate the search of complex proteoform (Fig. 1A). Basically, a SQPD is a portable and well-structured SQL database file (based on SQLite 3.x), which offers high performance for querying or sorting the data. The detailed database structure of SQPD is shown in Additional file 1: Table S1. Although there are three main databases that provide species-specific and ready-to-use protein sequences: RefSeq [9], Ensembl [10] and UniProt [11]. The UniProt database collects the most comprehensive protein sequence resources, and also provides detailed information about sequence variations, isoforms, mature processing and post-translational modifications. Thus, we chose UniProt as the main source of species-specific protein sequence databases for full proteoform annotations. As the controlled vocabularies (CVs) of proteoform features between UniProt and PEFF standards are slightly different, we then defined compatible terms for constructing SQPD database for UniProt (Additional file 1: Table S2). Alternatively, SeqWiz also supports the storage of protein sequences from other sources (such as NCBI, Ensembl or other customized sequences) without automatic annotation of proteoform information. Since protein sequences and features are already structured and stored table objects in SQLite based database, SQPD outperforms traditional text based FASTA or PEFF formats without the need for loading and parsing text contents. It can greatly decrease the computational cost and time for fetching protein sequences and features. Besides, it is also robust to large files (Additional file 2: Fig. S1A). In addition, SQPD is also easy-to-use programmatically according to the SQL language. The SET is based on the lightweight JSON data format, which is both human-readable and object-oriented (Fig. 1B). In complement to the SQPD format, the SET format is designed for generating subsets from the full database. A SET file only contains unique protein entries with minimal size and maximal flexibility (Additional file 2: Fig. S1B). Specifically, the SET files are used to store a selection of protein entries for the purpose of high-efficient narrowing-down database search, sequence filtering or results sharing.

Then, we mainly focused on the UniProt knowledgebase and developed a collection of tools for proteomic-centric sequence database management and analysis. In recent years, Python has been very popular in the analysis of biomedical data due to its simplicity and compatibility [12]. We thus chose Python (version: 3.x) as the main coding language, which already provides supports to SQLite3 and JSON for SQPD and SET data correspondingly. The required third-party modules are Requests (version: 2.x) for retrieving data via internet and Biopython (version: 1.7.x) for predicting physicochemical properties [13]. Moreover, we also use the wxPython (version: 4.x) widgets to provide graphical user interfaces (GUIs) across different operating system such as Windows, Linux and MacOS. All the tools are directly written in python scripts with standardized self-describing arguments for command line interface (CLI) usage. The GUI engine can generate visual shell with enhanced input widgets, automatic validations, pop-up hints and task monitoring for each tool. In addition to standalone tools, we also provided a basic module, named as “fastabase” to handle both text formatted files (FASTA or PEFF) and SQPD files for interactive coding or downstream analysis. Thus, the whole toolkit is friendly to both end-users as well as bioinformaticians. The SeqWiz toolkit is completely open source and follows the GNU General Public License (version 3.0). The source codes, stable distributions and testing files are available at https://github.com/fountao/protwiz/tree/main/seqwiz. To provide better documentations and collect debugs (or suggestions) timely, we also developed a specialized homepage for SeqWiz (http://websdoor.net/goto/seqwiz).

**Fig. 2:**
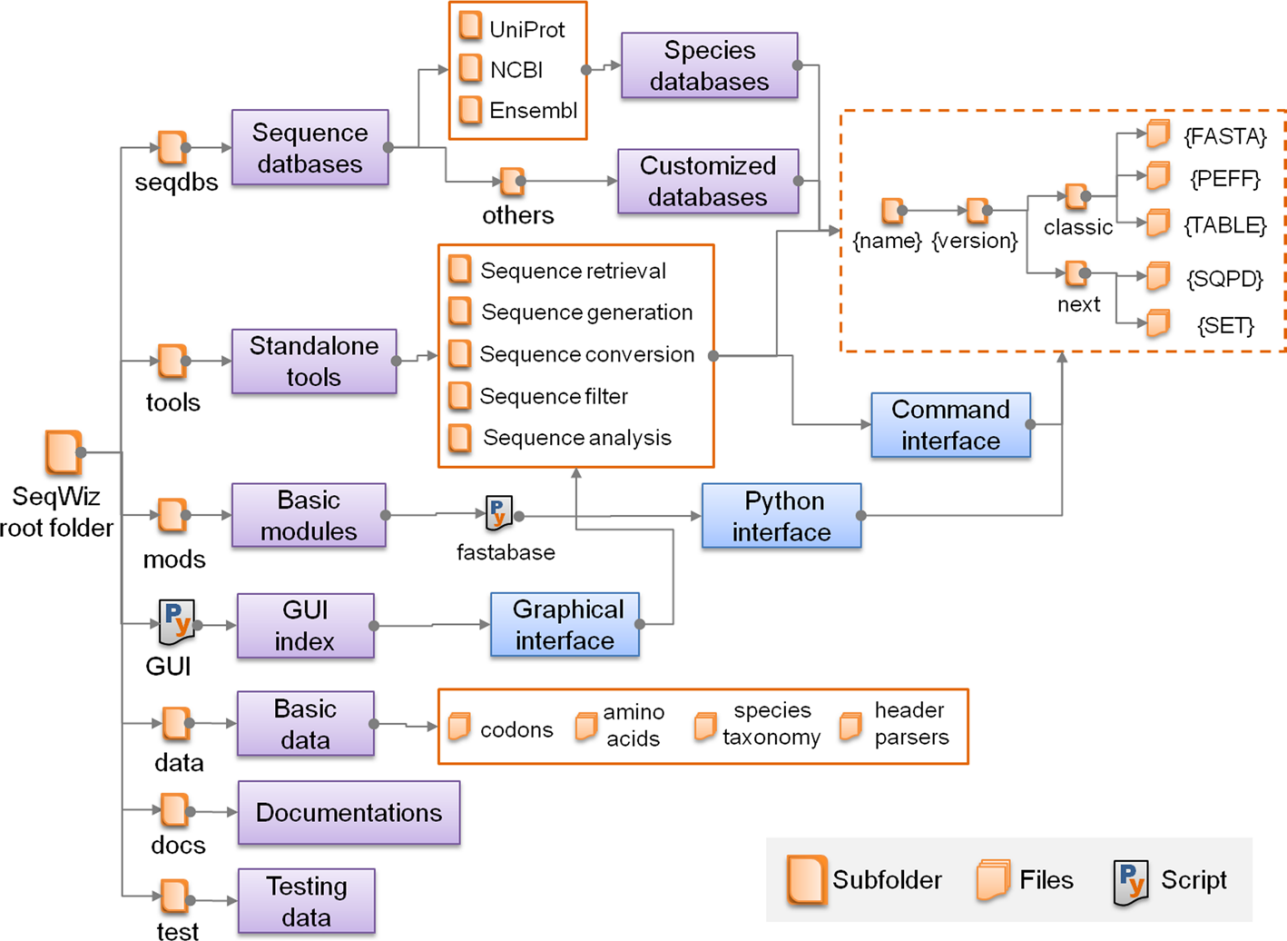
The main directory structure and functional categories of SeqWiz. SeqWiz contains six main subfolders for storing sequence databases, standalone tools, supporting modules, required data, documentations and test files. In addition, the GUI shell in the root folder provides an index interface to the tools

The main directory structure of SeqWiz is schemed Fig. 2. The “seqdbs” directory stores structured database files (including sequences, feature tables and subsets) for sequences derived from UniProt or other sources. Each database is organized and maintained by unique database name and different versions. Then all the data files are classified into “classic” (for traditional FASTA, PEFF and table files) and “next” subfolders (for the novel proposed SQPD and SET files). The “tools” folder contains the cataloged standalone tools. While the “mods” folder contains basic modules (including “fastabase”) for handling sequence files in interactive python environment or used for software development. The “data” folder contains predefined basic data (including codons, amino acids, species taxonomy, and header parsers) to support the basic module. The “docs” folder provides documentations for the usage of the tools as well as the basic module. The “test” folder offers sample files and test script for testing the functionalities. Finally, the “GUI.py” in the root folder provides an index interface for all tools.

## Results and discussion

To provide flexible and extensive features, SeqWiz is mainly designed as a collection of modularized functions with rich parameter settings. Hence, these tools could be used standalone for a specific mission, or combined in sequential processes to achieve complex tasks. These functions are classified into five categories, including sequence retrieval, sequence generation, sequence conversion, sequence filter and sequence analysis (Fig. 2). In the current release of SeqWiz (Version: 1.0), we offers a total of 18 tools with comprehensive functionalities (Additional file 1: Table S3), as described below:

1. Sequence retrieval (7 tools): First, UpSpecies could be used to search for the taxonomy ID as defined in UniProt for a specific species. Then, the taxonomy ID can be send to UpRetrieval to automatically download FASTA sequences and annotation table from UniProt. The default annotation table contains basic annotations (such as gene name and protein name) and proteoform annotations (such as sequence processes and post-translational modifications). The table can be used to construct feature table for the SQPD, follows the similar CVs defined in PEFF standards, In addition to the UniProt database, SeqWiz also provides NCBISpecies, NCBIRetrieval, EnsemblSpecies and EnsemblRetrieval tools to fetch the taxonomy and species specific sequences from the NCBI or Ensembl database correspondingly. However, due to the lack of adequate annotations, protein sequences from non-UniProt sources can only be converted into SQPD without features by default. However, the feature table can be generated manually as required. For other local sequences (such as contaminants or predicted sequences), the DbManage tool can be used to generate and update sequence database in the directory of “seqdbs”.
2. Sequence generation (3 tools): These tools are used to generate text based FASTA sequences for specific usages. First, SeqDecoy can be used to create reversed sequences for the evaluation of false discovery rates [14]. MatureSeq can generate mature forms (including processed chains, signal peptide, propeptide and transit peptide) of protein sequences for precise database search. The naming convention of a unique mature sequence is schemed in Additional file 2: Fig. S2A, which indicates the source protein ID, polypeptide type and truncated position range. Recent years, more and more evidences showed that small open reading frame (sORF) can be hidden in miRNA (primary sequences), lncRNA, and circRNA transcripts [15].The combination of transcriptomics and proteomics (or peptidomics) are proven to be highly efficient in screening of sORF encode peptides (SEPs) or microproteins [16, 17]. Thus, we also developed a bleeding-edge tool (SepFinder) to predict potential sORFs and SEPs based on the given RNA sequences, considering non-canonical initial codons and peptide length. The naming convention of a sORF or SEP is schemed in Additional file 2: Fig. S2B, which indicates the source transcript ID, frame index, codon usage, RNA type (liner RNA or circular RNA) and position range of ORF. For circular RNAs, SepFinder also reports the frequency number of crossing the junction site for a specific sORF.
3. Sequence conversion (3 tools): First, CheckSeq provides functions to check the formats of FASTA, PEFF, SQPD, SET and even a list of peptides. Then, UpConvert can be used to convert FASTA sequences into PEFF or SQPD format, considering the annotation table. Finally, the SeqConvert tool provides simple functions to convert file formats between different formats (FASTA, PEFF, SQPD, and SET).
4. Sequence filter (2 tools): These tools offer useful functions to select a list of intended entries. SeqFilter can be used to filter sequence features (mass, length or proteoform) or motif in SQPD file. While TabFilter can be used to filter the physicochemical properties in the annotated table file. To provide minimal file size, filter tools only generate SET files.
5. Sequence analysis (3 tools): These tools provide enhanced functions for proteomic-centric sequence analysis. SeqAnnotate can be used to calculate and predict protein properties, including sequence length, molecular weight, residue composition (singular or grouped), isoelectric point, physiological charge, reduced molar extinction, cystines molar extinction, aromaticity, instability and grand average of hydropathy. MotifCount can be used to calculate the occurrence of a list of sequence motifs. SeqWindow can extract the nearby residues for a list of peptides or sites.

To demonstrate the usage and application of SeqWiz toolkit and the basic module, we also provide four practical tutorials within the released package:

1. The first example is a command tutorial. It intends to create an improved FASTA sequences with mature sequences (as annotated by UniProt) for mouse proteins, which can be used to improve sequence coverage or to discovery more PTMs (Additional file 2: Fig. S3).
2. The second example is a GUI tutorial. Using a testing FASTA file of circRNA transcripts, it aims to generate a predicted protein sequence database of SEPs via unattended commands (Additional file 2: Fig. S4).
3. The third example is to create different subsets for mouse protein database, and to generate classic FASTA files from SET files for compatible usage (Additional file 2: Fig. S5).
4. The last example is to show the usage of the “fastabase” module for handling SQPD and PEFF files in the interactive python coding environment. A comparison of the running time further shows that SQPD works hundreds of times faster than the text-based PEFF file (Additional file 2: Fig. S6).

Admittedly, some of the basic functions of SeqWiz have already been implemented in other softwares such as PyUniProt or Pyteomics [18]. However, SeqWiz is well-designed with some novel features. Inspired by the PEFF standards, we proposed new formats (SQPD and SET) for local sequence database and subsets, which are well-structured and high-efficiency. SeqWiz also offers bleeding-edge tools for generating mature sequences and predict potential SEPs, which provides opportunities for reanalysis of “old data” as raised in our previously proposed framework of complementary proteomics [19]. Moreover, all the functions are designed as standalone tools, which can be easily combined as needed in an automatic workflow of proteomic data analysis. Currently, SeqWiz is only focused on primary sequence analysis. However, due to the flexibility and extensibility of SQPD format, other structural and functional features can also be integrated into the database programmatically to make the following bioinformatics analysis more convenient.

## Conclusions

In summary, we proposed modernized sequence formats for ready-to-use database search, instant sequence analysis and minimized data sharing based on SQLite and JSON formats. Then, the SeqWiz was developed as an open-source toolkit with comprehensive functions for protein sequence database management and analysis. Besides, it also provides basic developing module to handle both the conventional FASTA/PEFF formats and the novel proposed SQPD formats. Hence, SeqWiz is both friendly to end-users who are not familiar with coding (for preparing ready-to-use sequence databases) and bioinformaticians (for performing downstream sequence analysis or software development). The toolkit is also designed as a highly flexible and extensible platform, which supports additional plug-ins for sequence analysis and can be easily connected to a search engine via SQL language. Besides being a useful tool, SeqWiz project will promote the implementing of complementary proteomics for data renewal and proteoform analysis to achieve precision proteomics. Additionally, it can also drive the improvement of proteomic standardizations and the development of next-generation proteomic softwares.

## Supplementary Information

**Additional file 1**: **Table S1**. The table and column structure of SQPD database. **Table S2**. Mapping table of the standard vocabularies between SQPD and PEFF. **Table S3**. Summary of the tools and their functionalities

**Additional file 2**: **Fig. S1**. A. Comparison of the file size and loading time between PEFF and SQPD files. B. Comparison of the file size and loading time between FASTA and SET files. **Fig. S2**. The naming conventions of mature sequencesand small ORF encoded peptides. **Fig. S3**. The workflow for creating mature sequences for mouse proteins. **Fig. S4**. The workflow for predicting SEPs derived from circRNA transcripts. **Fig. S5**. The workflow for generating subsets and retrieving sequences. **Fig. S6**. A coding example for the usage of the “fastabase” module

## Data Availability

The datasets generated and/or analysed during the current study are available in the GitHub repository, https://github.com/fountao/protwiz/tree/main/seqwiz.

## Abbreviations

PSI: Proteomics standards initiative
PEFF: PSI extended FASTA format
GUI: Graphical user interface
CLI: Command line interface
sORF: Small open reading frame
SEPs: SORF encode peptides

## Availability and requirements

*Project name*: SeqWiz (a modularized toolkit for next-generation protein sequence database management and analysis). *Project home page*: http://websdoor.net/goto/seqwiz. *Operating system(s)*: Microsoft Windows, Mac OS X and macOS, and Linux or other unix-like systems with GTK2 or GTK3 libraries. *Programming language*: python 3.x. *Other requirements*: wxPython (4.x), Requests (2.x), Biopython (1.x). *License*: GNU GPL v3. *Any restrictions to use by non-academics*: license needed.
