# Supplementary material for: SeqWiz: a modularized toolkit for next-generation protein sequence database management and analysis
Source: BMC Bioinformatics. 2023 May 17;24:201. doi: 10.1186/s12859-023-05334-9 (PMC10189941; doi:10.1186/s12859-023-05334-9)
Supplement: Supplementary file 1 — Additional file 1: Table S1. The table and column structure of SQPD database. Table S2. Mapping table of the standard vocabularies between SQPD and PEFF. Table S3. Summary of the tools and their functionalities [file 12859_2023_5334_MOESM1_ESM.docx]

**Table S1. The table and column structure of SQPD database**

| Table | Column | Description |
| --- | --- | --- |
| basicinfo | idx | Internal indexing ID |
| basicinfo | DbName | Database name |
| basicinfo | DbVersion | Database version |
| basicinfo | DbDate | Database date (created) |
| basicinfo | DbSource | Database source |
| basicinfo | DbDescription | Database description |
| basicinfo | Prefix | Database prefix |
| basicinfo | NumberofEntries | Number of sequences |
| basicinfo | Conversion | Conversion note |
| basicinfo | SequenceType | Seqeuence note |
| custominfo | idx | Internal indexing ID |
| custominfo | class | Custome class (CustomKeyDef or GeneralComment) |
| custominfo | ckey | Custome key |
| custominfo | cdesc | Custome description |
| custominfo | cnote | Custome note |
| custominfo | bidx | Corresponding Database ID |
| sequence | idx | Internal indexing ID |
| sequence | uid | Unique ID |
| sequence | sequence | Protein sequence |
| sequence | bidx | Corresponding Database ID |
| features | idx | Internal indexing ID |
| features | uid | Unique ID |
| features | fkey | Feature key |
| features | fval | Feature value |
| features | fcls | Feature class |
| features | fsource | Feature source |
| features | fnote | Feature note |

***Note:** The “basicinfo” table defines the basic information (terms for standardized CVs) for a database, while the “custominfo” table stores customized information. The “sequence” table stores protein sequences, while the “features” table stores annotation information for each sequence.

**Table S2. Mapping table of the standard vocabularies between SQPD and PEFF**

| SQPD table | Column name in SQPD | Feature Key name in SQPD | PEFF Section | Key name in SQPD |
| --- | --- | --- | --- | --- |
| basicinfo | DbName | - | **File header** | DbName |
| basicinfo | DbVersion | - | **File header** | DbVersion |
| basicinfo | DbDate | - | **File header** | DbDate |
| basicinfo | DbSource | - | **File header** | DbSource |
| basicinfo | DbDescription | - | **File header** | DbDescription |
| basicinfo | Prefix | - | **File header** | Prefix |
| basicinfo | NumberofEntries | - | **File header** | NumberofEntries |
| basicinfo | Conversion | - | **File header** | Conversion |
| basicinfo | SequenceType | - | **File header** | SequenceType |
| custominfo | - | CustomKeyDef | **File header** | CustomKeyDef |
| custominfo | - | GeneralComment | **File header** | GeneralComment |
| sequences | uid | - | **Entry header** | DbUniqueId |
| sequences | sequence | - | **Entry sequence** | - |
| features | - | Length | **Entry header** | Length |
| features | - | PName | **Entry header** | PName |
| features | - | GName | **Entry header** | GName |
| features | - | TaxName | **Entry header** | TaxName |
| features | - | UpModRes | **Entry header** | ModRes |
| features | - | UpVariantSimple | **Entry header** | Variant |
| features | - | UpProcessed | **Entry header** | Processed |
| features | - | UpLinked | **Entry header** | ModRes? |

*Other feature names supported by SQPD are directly the same as defined in UniProt columns. See: <https://www.uniprot.org/help/uniprotkb_column_names>

**Table S3. Summary of the tools and their functionalities**

| Category | Tool name | Functions | Note |
| --- | --- | --- | --- |
| Sequence Retrieval | UpSpecies | Search or view taxonomy ID | Based on UniProt |
| Sequence Retrieval | UpRetrieval | Download species specific sequences and annotations, create structured database | Based on UniProt, support full annotations, options to create SQPD and perform features prediction |
| Sequence Retrieval | NCBISpecies | Search or view taxonomy ID | Based on NCBI |
| Sequence Retrieval | NCBIRetrieval | Download species specific sequences | Based on NCBI, options to create SQPD and perform features prediction |
| Sequence Retrieval | EnsemblSpecies | Search or view taxonomy ID | Based on Ensembl |
| Sequence Retrieval | EnsemblRetrieval | Download species specific sequences | Based on Ensembl, options to create SQPD and perform features prediction |
| Sequence Retrieval | DbManage | Create structured database from other sequence sources |  |
| Sequence Generation | MatureSeq | Generate mature forms of protein sequences | Based on UniProt annotations |
| Sequence Generation | SepFinder | Predict sORF and SEPs from transcript sequences | Supports both liner and circular RNAs |
| Sequence Generation | SeqDecoy | Generate decoy FASTA sequences |  |
| Sequence Conversion | CheckSeq | Check the format of FASTA, PEFF, SQPD, SET or PEPLIS | *PEPLIS: a list of peptide sequence |
| Sequence Conversion | UpConvert | Convert FASTA from UniProt to PEFF or SQPD | Based on UniProt, support full annotations |
| Sequence Conversion | SeqConvert | Simple format converter for non-UniProt sequences, from FASTA to |  |
| Sequence Filter | SeqFilter | Sequence filter to generate a SET list from SQPD |  |
| Sequence Filter | TabFilter | Table filter to generate a SET list | *Recommend to use the result table from SeqAnnotate |
| Sequence Analysis | SeqAnnotate | Sequence statistics for singular or grouped residues; calculation or prediction of physicochemical properties, including: isoelectric point, physiological charge, reduced molar extinction, cystines molar extinction, aromaticity, instability and grand average of hydropathy. | *Require the biopython package |
| Sequence Analysis | MotifCount | Motif statistic for sequence files |  |
| Sequence Analysis | SeqWindow | Sequence window extraction from a position table (for a list of sites or peptides) |  |
