# Supplementary material for: SeqWiz: a modularized toolkit for next-generation protein sequence database management and analysis
Source: BMC Bioinformatics. 2023 May 17;24:201. doi: 10.1186/s12859-023-05334-9 (PMC10189941; doi:10.1186/s12859-023-05334-9)

**Figure S1.** A. Comparison of the file size and loading time between PEFF and SQPD files. B. Comparison of the file size and loading time between FASTA and SET files.


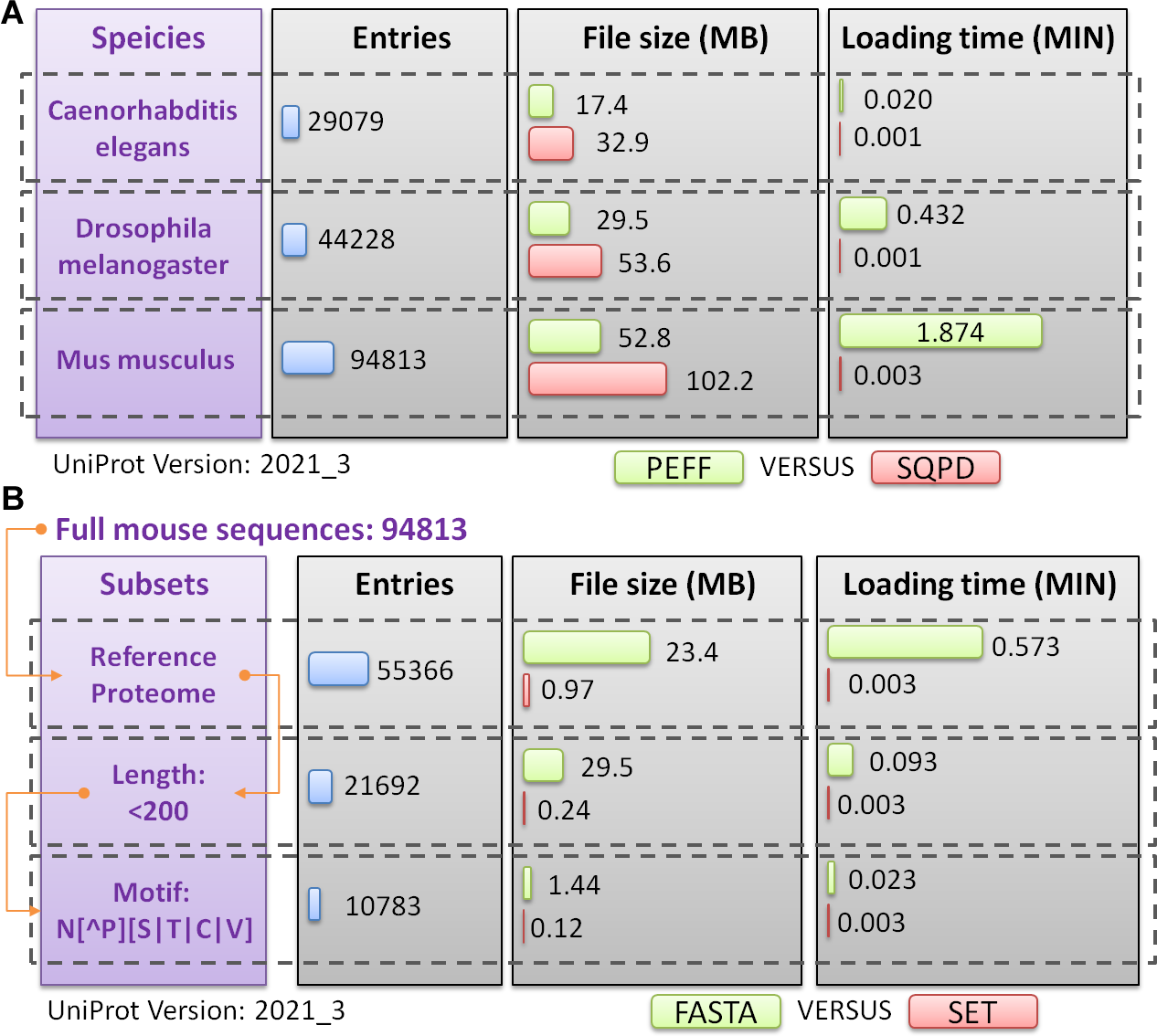


**Figure S2.** The naming conventions of mature sequences (A) and small ORF encoded peptides (B).


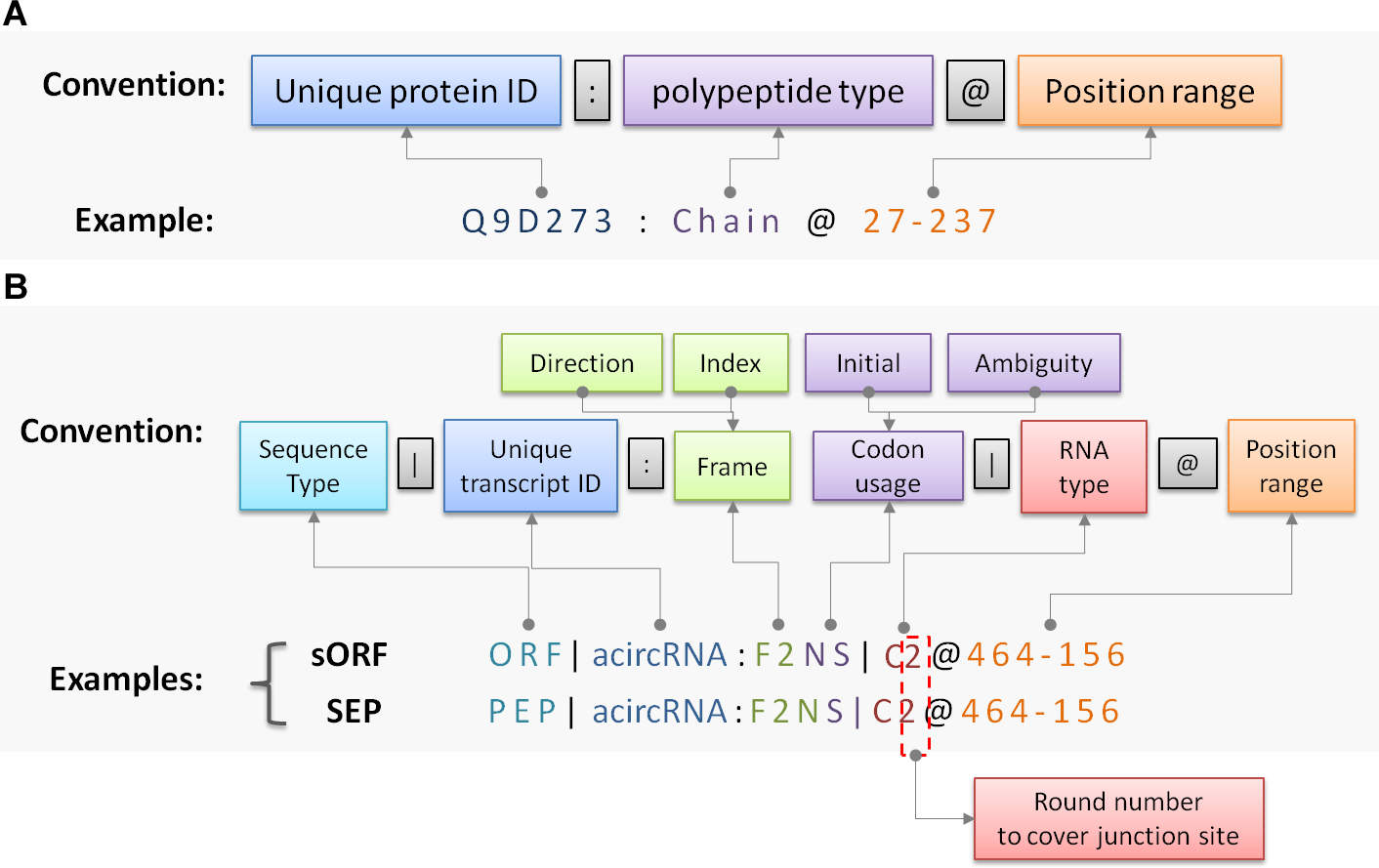


**Figure S3.** The workflow for creating mature sequences for mouse proteins


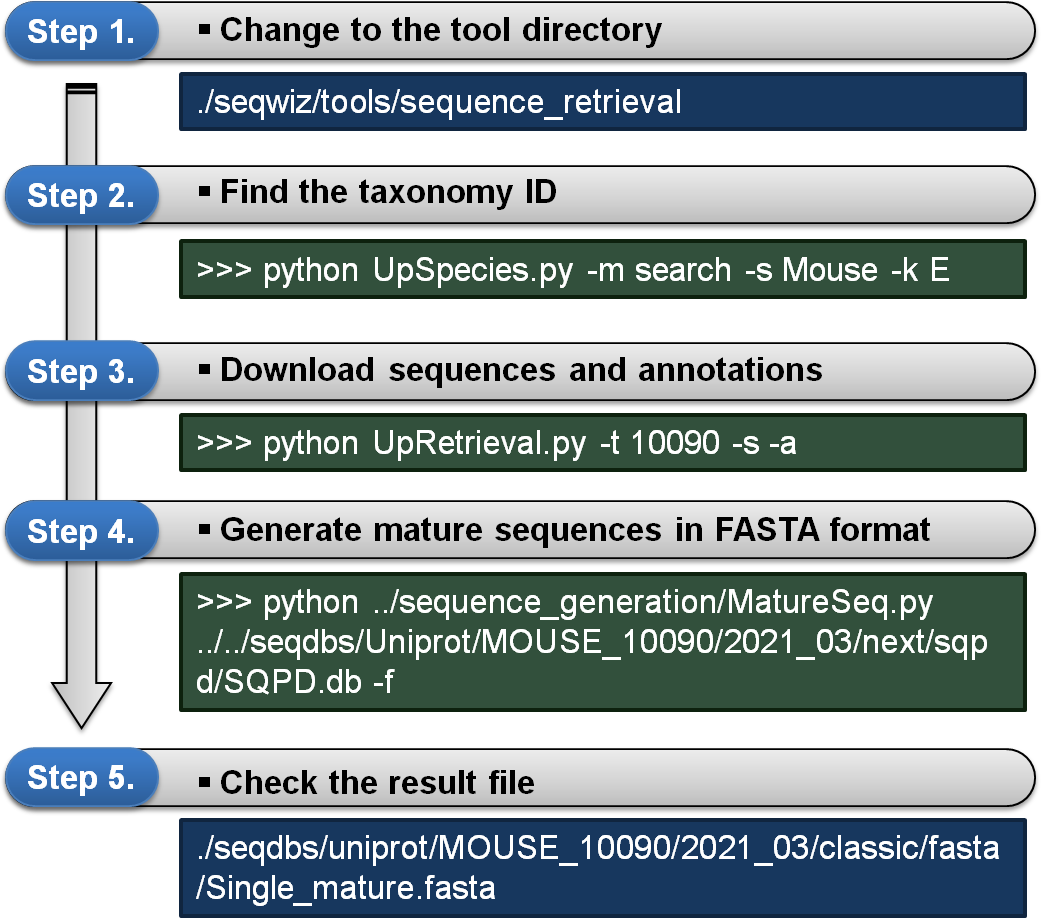


**Figure S4.** The workflow for predicting SEPs derived from lncRNA transcripts


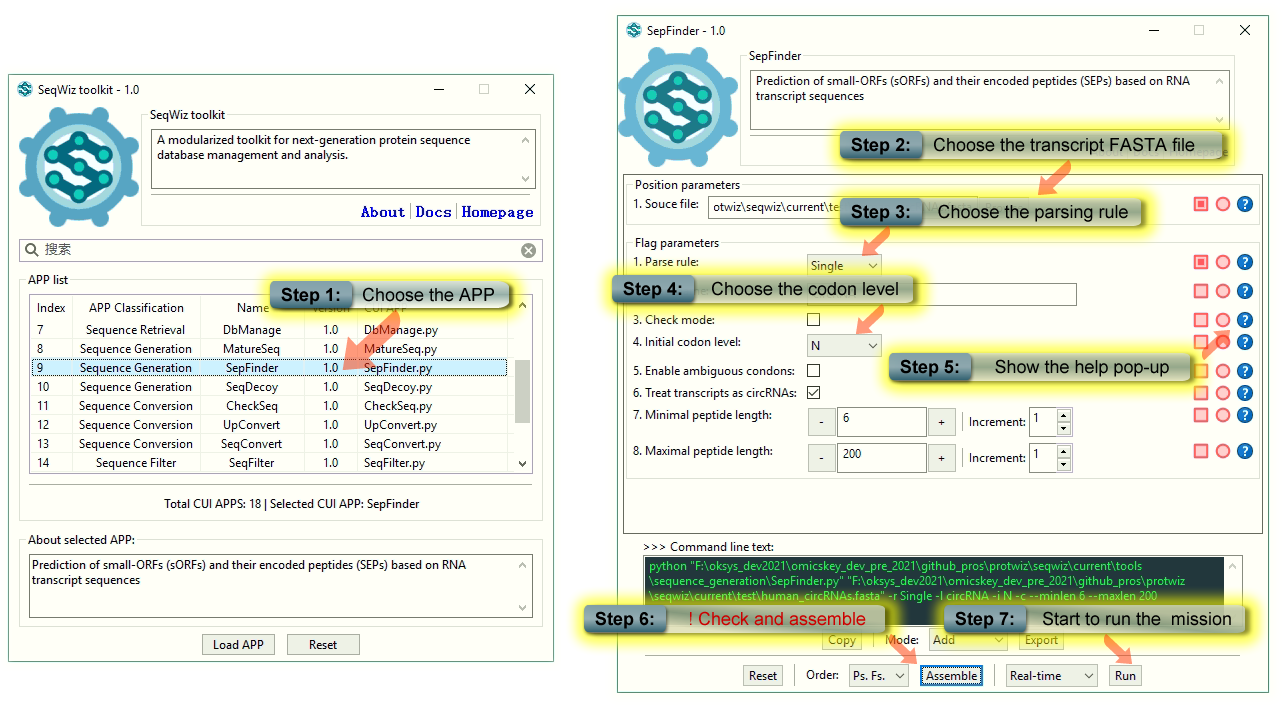


**Figure S5.** The workflow for generating subsets and retrieving sequences


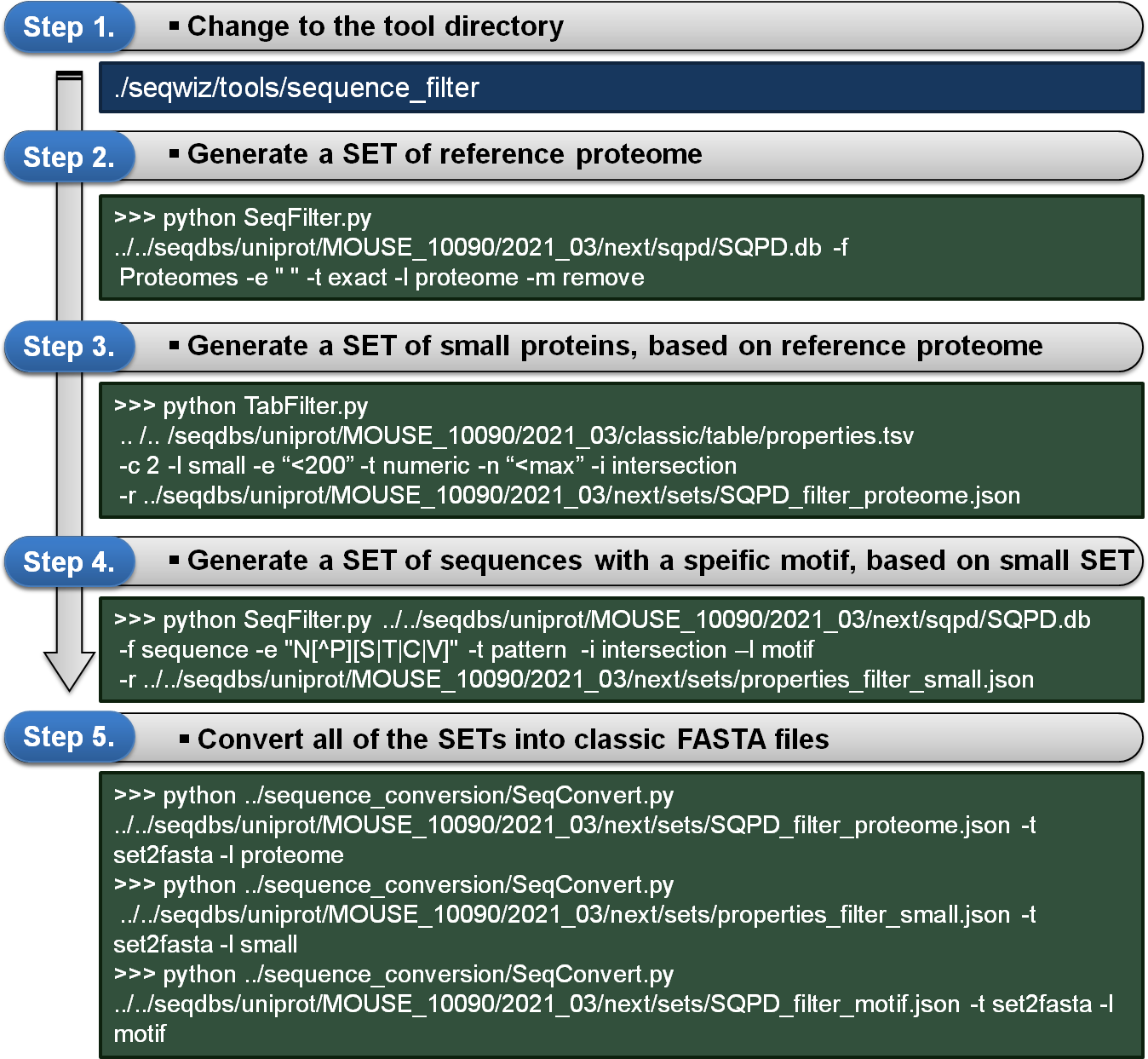


**Figure S6.** A coding example for the usage of the “fastabase” module


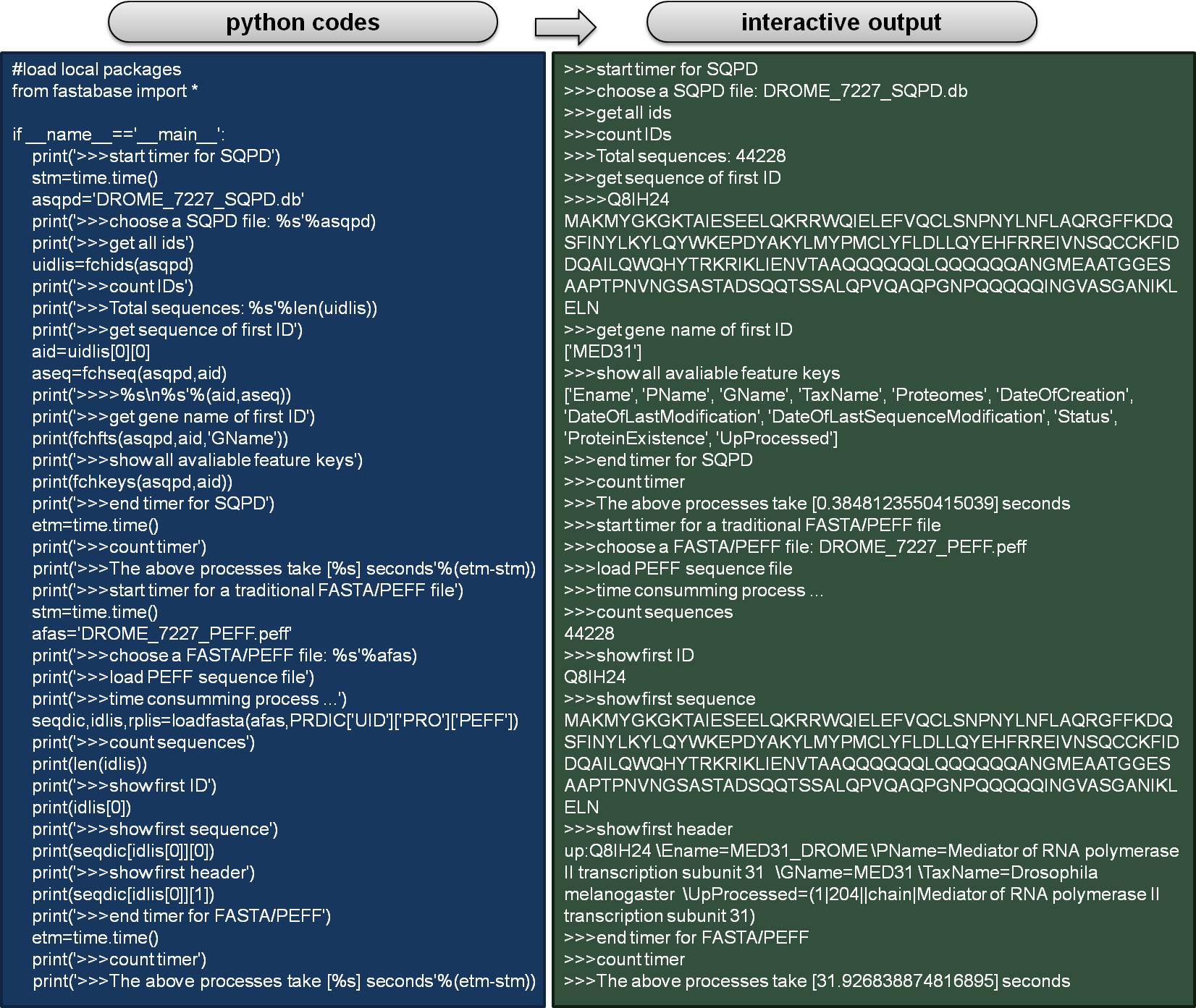

Supplement: Supplementary file 2 — Additional file 2: Fig. S1. A. Comparison of the file size and loading time between PEFF and SQPD files. B. Comparison of the file size and loading time between FASTA and SET files. Fig. S2. The naming conventions of mature sequencesand small ORF encoded peptides. Fig. S3. The workflow for creating mature sequences for mouse proteins. Fig. S4. The workflow for predicting SEPs derived from circRNA transcripts. Fig. S5. The workflow for generating subsets and retrieving sequences. Fig. S6. A coding example for the usage of the “fastabase” module [file 12859_2023_5334_MOESM2_ESM.docx]
